# Some Glycoproteins Expressed on the Surface of Immune Cells and Cytokine Plasma Levels Can Be Used as Potential Biomarkers in Patients with Colorectal Cancer

**DOI:** 10.3390/biom14101314

**Published:** 2024-10-16

**Authors:** Tsvetelina Batsalova, Denitsa Uzunova, Gergana Chavdarova, Tatyana Apostolova, Balik Dzhambazov

**Affiliations:** 1 Faculty of Biology, Paisii Hilendarski University of Plovdiv, 24 Tsar Assen Str., 4000 Plovdiv, Bulgaria; tsvetelina@uni-plovdiv.bg (T.B.); denitsa_uzunova@uni-plovdiv.bg (D.U.); 2 Medical Institute of Ministry of Interior, 79, Skobelev Blvd., 1606 Sofia, Bulgaria; 3 University Hospital “Pulmed”, 1A, Perushtitsa Str., 4002 Plovdiv, Bulgaria

**Keywords:** colorectal cancer, biomarkers, glycoproteins, cytokines

## Abstract

Colorectal cancer (CRC) is a leading cause of mortality worldwide. Its incidence holds a major position among the most common life-threatening diseases. Hence, the early identification and precise characterization of disease activity based on proper biomarkers are of utmost importance for therapeutic strategy and patient survival. The identification of new biomarkers for colorectal cancer or disease-specific levels/combinations of biomarkers will significantly contribute to precise diagnosis and improved personalized treatment of patients. Therefore, the present study aims to identify colorectal cancer-specific immunological biomarkers. The plasma levels of several cytokines (interleukin-1β /IL-1β/, IL-2, IL-4, IL-10, IL-12, IL-15, TGFβ and IFNγ) of 20 patients with colorectal cancer and 21 healthy individuals were determined by ELISA. The expression of several types of glycoproteins on the surface of peripheral blood leukocytes isolated from CRC patients and healthy volunteers was evaluated by flow cytometry. Correlations between cytokine levels and cell surface glycoprotein expression were analyzed. The obtained results demonstrated significantly elevated levels of CD80, CD86, CD279 and CD274 expressing leukocyte populations in the cancer patient group, while the numbers of NK cells and CD8- and CD25-positive cells were decreased. Based on these data and the correlations with cytokine levels, it can be concluded that CD25, CD80, CD86, CD274 and CD279 glycoproteins combined with specific plasma levels of IL-1β, IL-2, IL-15 and TGFβ could represent potential biomarkers for colorectal cancer.

## 1. Introduction

Colorectal cancer (CRC) is one of the most common malignant diseases. Statistically, it ranks third among all cancer types concerning both incidence and mortality [1,2]. One of the main reasons for the high morbidity and mortality caused by CRC is the long and often asymptomatic initial stage of the disease, which delays diagnosis and timely treatment. Early diagnosis is an important prerequisite for adequate therapy and CRC patient survival. Recent advances in biomarker research based on genomics, transcriptomics, proteomics and metabolomics have pointed to new strategies for disease detection and the prediction of treatment outcomes [3]. The identification of certain gene mutations (adenomatous polyposis coli (*APC*), *Kirsten RAS* proto-oncogene GTPase (*KRAS*), *B-Raf* proto-oncogene serine/threonine kinase (*BRAF*), etc.), as well as the characterization of microsatellite instability, has been included in disease profiling and therapeutic planning mainly for patients with metastatic disease [3,4]. However, there are still few validated biomarkers in clinical practice, and the search for new CRC-specific diagnostic, prognostic and predictive disease markers continues.

With the advent of immunotherapy and the increasing application of liquid biopsy that allows minimally invasive isolation of peripheral blood cells, circulating tumor cells, specific proteins, micro-RNAs, long noncoding RNAs and circulating tumor DNA, new possibilities for improved diagnosis and personalized medicine have emerged [3,5,6]. In particular, analyses of the immunomodulatory cell surface and circulating molecule profiles have already shown indispensable potential for the identification of new biomarkers for CRC [7,8,9,10,11].

A link between inflammation and carcinogenesis has been established based on the reported impact of inflammatory cytokines on tumor growth and progression [12]. It has been confirmed that inflammation contributes to CRC development and circulatory cytokines could regulate the immune response within the tumor microenvironment [13,14]. The delicate balance between pro-inflammatory and anti-inflammatory cytokines plays a crucial role in the organisms’ homeostasis. If it is disturbed, CRC could develop [7]. Aberrant cytokine expression induces inflammation in CRC and drives immunosuppression, resistance to therapeutics and metastasis during CRC progression [14]. Significant differences in the cytokine profile of CRC patient groups compared to healthy controls have been shown in a number of studies [12,15,16,17,18]. Therefore, predictive models based on multiple cytokine measurements have been proposed, and these signaling molecules appear to be promising candidate biomarkers for CRC. However, the investigations into cytokine levels varied significantly in terms of patient group characteristics and the number and type of analyzed cytokines, establishing a need for validation in larger trials. Therefore, to define a more specific biomarker model for CRC, a correlation between different cytokine levels and immunomodulatory molecules involved in immune cell activation/suppression and cytokine production should be considered.

In this study, we investigated the serum levels of several cytokines associated with CRC pathogenesis and the expression of immunomodulatory markers on peripheral leukocytes in CRC patients with stage IV disease progression compared to healthy controls and their specific correlations with CRC.

## 2. Materials and Methods

### 2.1. Participants in the Study

Two groups of individuals participated in the study—20 patients with stage IV colorectal cancer undergoing chemotherapy and regular medical examinations (age range 45–80 years) and 21 healthy volunteers (age range 40–72 years). Blood sampling was carried out according to The Code of Ethics of the World Medical Association (Declaration of Helsinki) approved for experiments involving humans.

All participants were informed about the aims of the study and signed a written consent form prior to sample collection. The experiment was approved by the Local Ethical Committee at Paisii Hilendarski University of Plovdiv, Bulgaria (protocol No. 5 from 10 June 2020), and implemented in accordance with the Declaration of Helsinki.

### 2.2. Blood Collection and Sample Preparation

Venous blood was obtained from all participants in the study and collected in BD Vacutainer^®^ K2 EDTA tubes (Becton, Dickinson and Company, NJ, USA). The samples were centrifuged at 1000× *g* for 15 min. Then, the plasma was separated from the cell pellet and stored at −20 °C until analysis of cytokine levels. The blood cell pellet was further processed in order to isolate leukocytes. This was achieved by gentle suspending of the cell pellet in 0.84% NH_4_Cl buffer (Merck KGaA, Darmstadt, Germany) and incubation at room temperature for 8–10 min. After this incubation, the erythrocytes were lysed, the samples were centrifuged at 1000× *g* for 15 min and the leucocytes were resuspended in Dulbecco’s phosphate-buffered saline (DPBS) (Merck KGaA, Darmstadt, Germany). After a second round of centrifugation under the same conditions, the cells were resuspended in FACS buffer (DPBS, containing 5% fetal bovine serum and 0.05% NaN_3_, all from Merck KGaA, Darmstadt, Germany) and used for flow cytometry staining and analyses.

### 2.3. Cytokine Evaluations

ELISA kits for IL-1β, IL-2, IL-10, IL-12, IL-15, TGFb1, IFNγ (all purchased from Diaclone SAS, Besancon Cedex, France), IL-4 and IL-13 (provided by BioLegend Inc., San Diego, CA, USA) were used for the evaluation of cytokine levels in blood plasma samples. The assays were performed according to the protocol provided by the manufacturer. Optical density (OD) measurements were performed using a SpectraMax i3x spectrophotometer (Molecular Devices, San Jose, CA, USA). The OD value for each sample was blank-corrected and used to calculate cytokine concentration based on the standard curve that included 6 different concentrations of the measured cytokine (for IL-1β, IL-2, IL-10, IL-12, IL-15, TGFb1 and IFNγ) or 7 different concentrations of the analyzed molecule (for IL-4 and IL-13).

### 2.4. Flow Cytometry Analyses

Leukocyte samples were incubated with fluorochrome-labeled antibodies specific to the markers CD3, CD4, CD8, CD16, CD20, CD25, CD28, CD56, CD152, CD273, CD274 and CD279 (BD Pharmingen™, BD Biosciences, Franklin Lakes, NJ, USA) and incubated for 20 min in a dark environment. After that, the cells were washed two times in FACS buffer and analyzed on a flow cytometer instrument, Cytomics FC500 (Beckman Coulter Inc., Life Sciences, Indianapolis, IN, USA). Different leukocyte populations were evaluated and compared between the control and the CRC group.

### 2.5. Statistics

The cytokine concentrations and flow cytometry data were determined to be not normally distributed, and, thus, nonparametric tests were used for statistical analyses. The Mann–Whitney *U* test and Spearman rank correlation were applied using StatView software 5.0 (SAS Institute Inc., Cary, NC, USA). At the value of *p* < 0.05, the results were considered statistically significant.

## 3. Results

### 3.1. Levels of Circulating Inflammatory Cytokines in Stage IV CRC Patients

**Figure 1:**
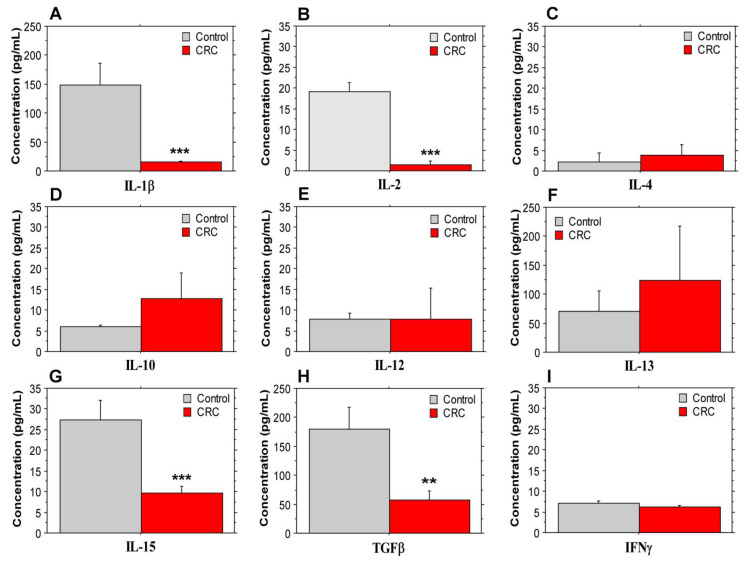
Plasma concentrations of inflammatory cytokines in stage IV CRC patients and control healthy volunteers. Levels of IL-1β (**A**), IL-2 (**B**), IL-4 (**C**), IL-10 (**D**), IL-12 (**E**), IL-13 (**F**), IL-15 (**G**), TGFβ (**H**) and IFNγ (**I**). Data represent ± standard error of the mean (±SEM). A Mann–Whitney *U* test was used for statistical analyses. ** *p* < 0.01, *** *p* < 0.001.

The concentration of several cytokines (IL-1β, IL-2, IL-4, IL-12, IL-13, L-15, TGFβ and IFNγ) involved in CRC pathogenesis [8] was evaluated in blood plasma samples from stage IV patients and healthy controls. As shown in Figure 1, we observed a significant decrease in the plasma concentration of Il-1β, IL-2, IL-15 and TGFβ in CRC patients. IL-12 (Figure 1E) and IFNγ (Figure 1I) levels did not differ between the CRC and control groups. A tendency for an increased concentration of IL-4, IL-10 and IL-13 in blood plasma of patients with metastatic disease was evident (Figure 1C,D,F). Although not significant (possibly due to the higher variation between the examined individuals), our results are concordant with the well-established CRC-promoting role of the above-mentioned three cytokines [8,15,16]. On the other hand, the markedly reduced peripheral concentration of IL-2, IL-15 and TGFβ in the CRC (Figure 1B,G,H) group could be explained by the advanced disease stage of the patients and the reported CRC-suppressive phenotype [8] of these cytokines or their ambiguous role (specifically for TGFβ); disease progression and chemotherapeutic treatment are logically associated with lower levels of signaling molecules with a potential anti-tumor role. The significantly lower levels of IL-1β in the CRC group (Figure 1A) could be due to the overall immunological state of the patients and the predominant processes of immune response suppression or exhaustion, as could be suggested based on the data in the next subsection. However, further studies are needed to support this hypothesis.

### 3.2. Expression of Some Surface Glycoproteins on Immune Cells Differ in CRC Patients with Metastatic Disease Compared to Healthy Individuals

**Figure 2:**
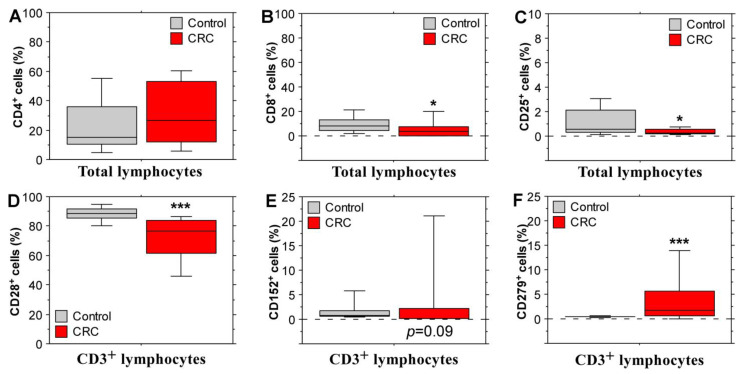
Flow cytometry analyses of the expression of T-cell-specific markers on peripheral blood leukocytes derived from stage IV CRC patients and healthy individuals. CD4^+^ T cells (**A**), CD8^+^ T cells (**B**), CD25^+^ T cells (**C**), CD28^+^ T cells (**D**), CD152^+^ T cells (**E**), and CD279^+^ T cells (**F**). The results are presented as ±SEM. Statistical significance was defined by the Mann–Whitney *U* test. * *p* < 0.05, *** *p* < 0.001.

Immunophenotyping analyses with peripheral blood leukocytes were performed in order to define the expression of certain immune cell surface glycoproteins and compare their level between stage IV CRC patients and healthy controls. The main lymphocyte populations were assessed based on expression of CD3, CD4, CD8 (all three markers are specific to T lymphocytes), CD 20 (a B cell marker), CD16 and CD56 (markers for NK cells). In addition, we evaluated the expression of the activation marker CD25 and immunomodulatory surface proteins—CD28, which exerts a positive role for T cell activation [19]; CD152 (cytotoxic T-lymphocyte-associated antigen 4 /CTLA-4/) and CD279 (programmed death 1 /PD-1/), which participate in the negative regulation of T cell immune function; and several B7 family molecules (CD80, CD86, CD273 /PD-L2/ and CD274 /PD-L1/). This selection of cell surface glycoproteins allowed for the basic evaluation of the activation or inhibition state of peripheral immune cells in patients with stage IV CRC. The results displayed in Figure 2 highlight important differences between the control group and the patient group. CD8^+^ lymphocyte populations were significantly diminished in the CRC patients (Figure 2B). The mean percentage of lymphocytes expressing the activation marker CD25 was also reduced (Figure 2C) in the patient group together with CD28 expression on T cells (Figure 2D). These data point to a reduced lymphocyte activation state and decreased levels of cytotoxic T lymphocytes in the peripheral blood of stage IV CRC patients, which is concordant with the advanced disease stage and inability of the immune system to resist cancer cells. Moreover, immunomodulatory markers with inhibitory activity were elevated in the patient group; a tendency for an increased expression of CTLA-4 (CD152) surface glycoprotein (Figure 2E) and a significantly higher percentage of CD279^+^ (PD-1^+^) T cells (Figure 2F) were observed. No differences between the levels of CD4^+^ lymphocytes were observed.

**Figure 3:**
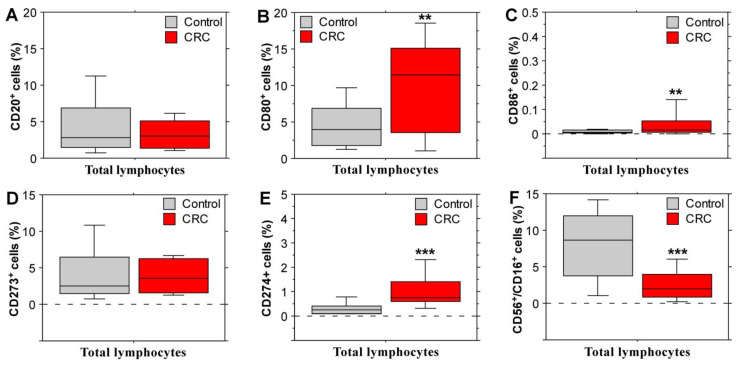
Levels of CD20^+^ B cells, NK cells and expression of some B7 family molecules in the peripheral blood of stage IV CRC patients and healthy controls. Percentage of CD20^+^ B cells (**A**), CD80^+^ B cells (**B**), CD86^+^ B cells (**C**), CD273^+^ lymphocytes (**D**), CD274^+^ lymphocytes (**E**), and CD56^+^/CD16^+^ NK cells (**F**). Data are shown as ±SEM. Statistical analyses were performed using the Mann–Whitney *U* test; ** *p* < 0.01, *** *p* < 0.001.

The B cell populations (Figure 3A) and CD273^+^-lymphocyte (Figure 3D) percentages did not differ between stage IV CRC and control subjects. However, the populations of CD80^+^, CD86^+^ and CD274^+^ lymphocytes (Figure 3B,C,E) were significantly increased in the peripheral blood of CRC patients. These glycoproteins from the B7 family serve as ligands for CTLA-4 (CD80/86) and PD-1 (CD274). Considering the elevated percentages of CD152^+^ and CD279^+^ lymphocytes, these data again support an overall immunosuppressive or exhausted (particularly in the case of PD-1 signaling) state.

Differences were detected in the levels of T lymphocyte-specific surface glycoprotein expression but also in the NK cell population, which was reduced in the group with metastatic CRC (Figure 3F). Considering the fact that NK lymphocytes represent a group of innate immune cells able to eliminate cancer cells without previous sensitization [20], these data support the suggestion of a suppressed state of the peripheral immune responses that favor CRC development.

### 3.3. Plasma Levels of IL-2 and IL-15 Correlate with Some Inflammatory Cytokines

**Figure 4:**
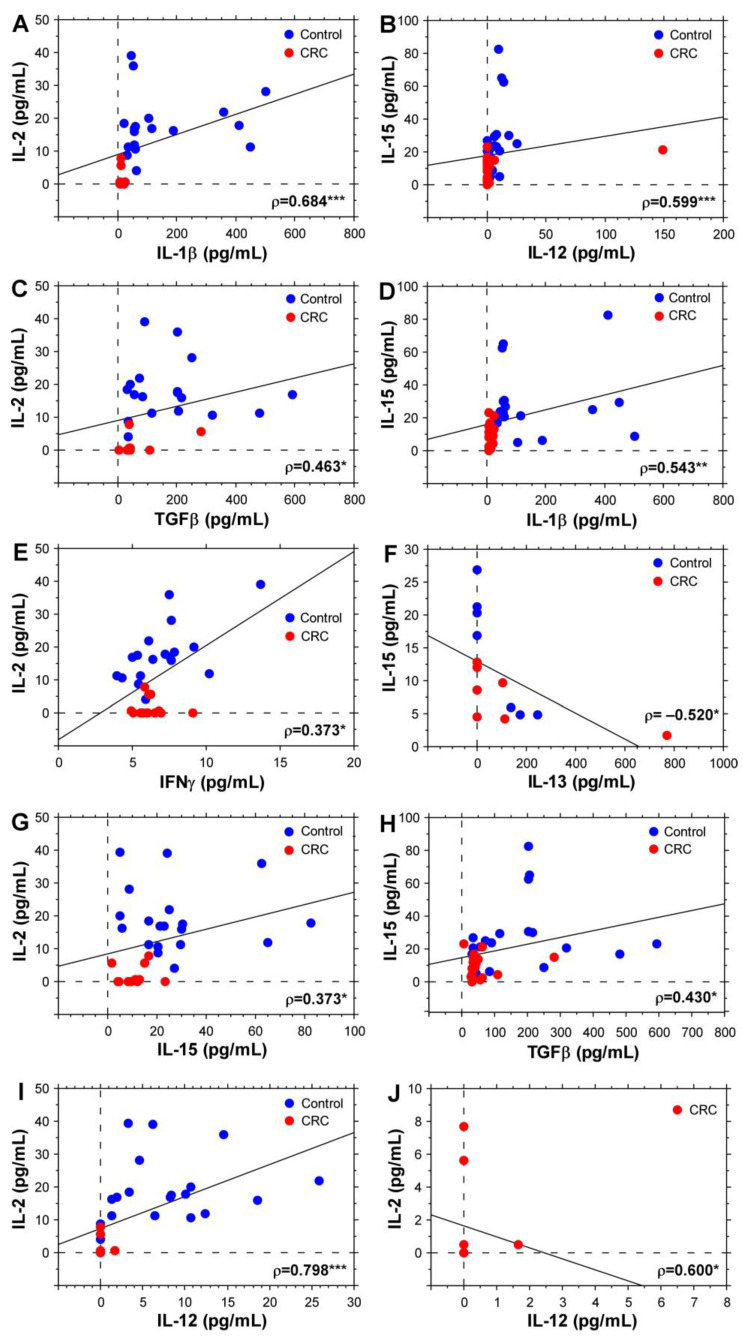
Correlations between IL-2 and IL-15 plasma concentrations with other cytokines. First column: IL-2 vs. IL-1β (**A**), IL-2 vs. TGFβ (**C**), IL-2 vs. IFNγ (**E**), IL-2 vs. IL-15 (**G**), IL-2 vs. IL-12 (**I**); Second column: IL-15 vs. IL-12 (**B**), IL-15 vs. IL-1β (**D**), IL-15 vs. IL-13 (**F**), IL-15 vs. TGFβ (**H**), and IL-2 vs. IL-12 only for CRC patients (**J**). The charts indicate Spearman’s rank correlation coefficient (ρ). * *p* < 0.05, ** *p* < 0.01, *** *p* < 0.001.

To further investigate the relationships between different inflammatory cytokines and strengthen their importance as potential biomarkers for CRC, we performed correlation analyses. Figure 4 shows the moderate and strong correlations observed for the measured cytokines. The aim of these analyses was to define general correlations using data from both study groups. The IL-2 plasma concentration showed a strong correlation with IL-1β and IL-12 levels (Figure 4A,I). The IL-2/IL-12 correlation coefficient retained a significant value even if data for healthy individuals were excluded (Figure 4J).

IL-2 and IL-15 plasma levels correlated positively with the concentration of other cytokines with a reported CRC-suppressive role—IFNγ, TGFβ and IL-12 (Figure 4B,C,E,G–I) [8]. IL-15 plasma levels correlated negatively with IL-13, supporting the CRC-promoting role of IL-13 (Figure 4F). Interestingly, a strong positive correlation was observed between IL-2 and IL-1β (Figure 4A), as well as a moderate positive correlation between IL-15 and IL-1β (Figure 4D). These data suggest a possible dual role of IL-1β involving both a CRC-promoting and CRC-suppressing phenotype depending on the disease stage and study group specifics. This association needs to be further investigated.

### 3.4. Stage IV CRC-Specific Correlations between Plasma Levels of Some Inflammatory Cytokines and Surface Glycoproteins on Immune Cells

**Figure 5:**
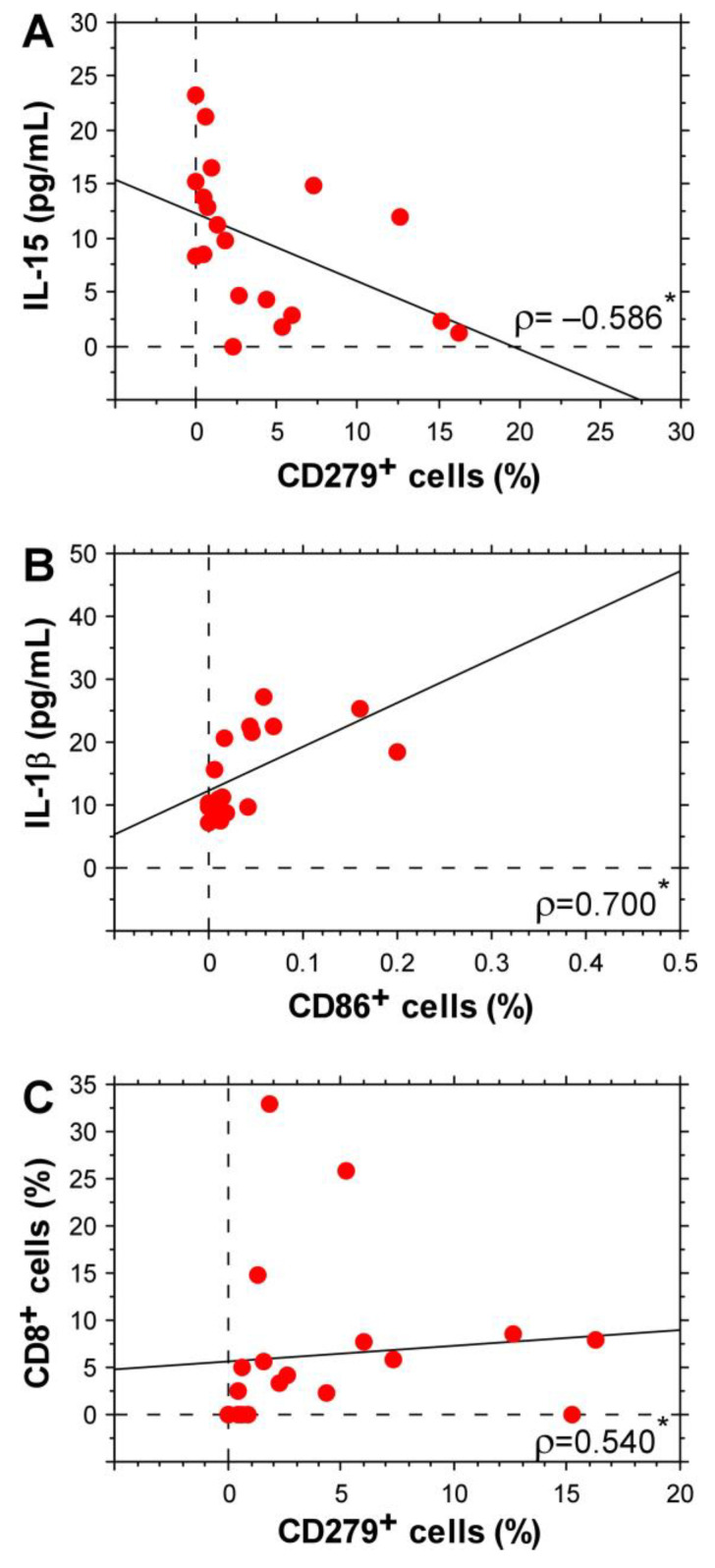
Correlations between circulating cytokines and immunomodulatory molecule expression levels determined for the stage IV CRC patient group. IL-15 vs. CD279^+^ cells (**A**), IL-1β vs. CD86^+^ cells (**B**) and CD8^+^ vs. CD279^+^ cells (**C**). The graphs show Spearman’s rank correlation coefficient (ρ). * *p* < 0.05.

Our studies demonstrated significantly different levels of certain cytokines and surface glycoproteins in the CRC group of patients with stage IV disease undergoing chemotherapeutic treatment. Therefore, we proceeded our analyses with correlations between the cytokine levels and expression of immunomodulatory glycoproteins that are specific to CRC patients and were not observed in the general group that included all samples. Figure 5A shows that IL-15 correlated negatively with CD279 expression on T cells. These results support the opposing role of the two molecules in CRC. IL-1β levels showed a strong positive correlation with CD86 expression on peripheral blood leukocytes (Figure 5B). This could be associated with the role of IL-1 signaling in antigen-presenting cell differentiation involving the upregulation of costimulatory molecules [21].

Another specific positive correlation for the CRC patient group was determined for the populations of CD8^+^ and CD279^+^ cells (Figure 5C). Further studies are needed to confirm whether this result is due to the enhanced expression of PD-1 on CD8^+^ T lymphocytes. A correlation between the CD279^+^ population and CD4^+^ T cells was not found.

## 4. Discussion

Colorectal cancer is one of the most common malignancies associated with high morbidity and mortality worldwide [1]. Early diagnosis of the disease could significantly improve patient treatment outcomes and survival. The identification of biomarkers or CRC-specific combinations of biomarkers is crucial for adequate diagnosis, patient stratification and personalized treatment [4]. Although genetic and epigenetic markers have been identified [6], CRC is a heterogenic type of disease [22], and, thus, there is still a need for more specific biomarkers that will facilitate the choice of treatment, especially for patients with advanced metastatic disease, improving their quality of life and overall survival.

The immune system plays major role in the fight against cancer cells [11], and immunological studies have provided key strategies for new cancer therapeutics. Consequently, it is reasonable to suggest that immune cell surface molecule expression and circulating signaling molecules (cytokines) with aberrations in their levels could provide new insights into disease biomarker discovery. Moreover, CRC is characterized by a strong inflammatory component; the disease frequently develops as a consequence of an inflammatory disorder [23], and, hence, cytokines and immune cell surface glycoproteins are potential candidates for new diagnostic, predictive and prognostic biomarkers. In line with this notion, our study was focused on the examination of immune cell surface markers and immunomodulatory molecules on peripheral blood leukocytes, as well as circulating cytokine levels, in a small cohort of stage IV CRC patients with the aim of highlighting potential candidate biomarkers. Several recent studies have reported altered levels/expression of different cytokines in CRC patients [15,16,17,18,24,25]. The results in some of them vary for specific interleukins, which could be due to patient and control group characteristics—stage of disease, number of examined individuals, applied therapy, etc. Our results for IL-4, IL-10 and IL-13 levels are concordant with those of previous studies [9,15,18,26,27], while the data on IFNγ levels differ between reports. Farc et al. confirmed our finding of a lack of difference in IFNγ serum concentrations between CRC patients and healthy controls [17]. However, a recent study of a Tunisian cohort indicated altered serum levels of IFNγ and IL-12 [24], which could be due to higher variability of the studied CRC group. In support of this suggestion, Li et al. showed no significant difference in IFNγ levels between CRC patients at stage IV and healthy controls, but a tendency for reduced cytokine levels could be noted in stage IV patients [18]. Urbiola-Salvador et al. demonstrated a significantly lowered IFNγ concentration in late-stage CRC patients [25]. In addition, Li et al. compared cytokine and chemokine levels in patients with different disease stages (I, II, III and IV) and showed that the levels of IFNγ and IL-4 could change considerably [18]. The IFNγ concentration in the peripheral blood of stage II and IIIB-C was significantly lower than that in healthy controls, while, during stage I and IIIA, the cytokine levels did not differ from those of the control. The IL-4 concentration was markedly increased in stage IV patients, while stage I, II, IIIA and IIIB-C patients did not differ considerably compared to the control group [18].

We detected a decreased concentration of IL-2, IL-15 and TGFβ in the CRC group, which is expected considering the previously characterized CRC-suppressive action of these cytokines [8]. The anti-tumor properties of IL-2 and IL-15 have been reported [28,29], and IL-15 has been highlighted as a potential biomarker and therapeutic target in CRC [9]. An ambiguous role could be defined for TGFβ [8,9]. Our data indicate that the concentration of this cytokine is markedly reduced in the blood plasma of stage IV CRC patients. In our studies, the TGFβ plasma levels correlated with IL-2 and IL-15, which could support a CRC-suppressive role for TGFβ.

Regarding IL-1β, we obtained a surprising result—a significantly lower plasma cytokine concentration in the CRC group compared to the control group. Due to the well-characterized CRC-promoting role of IL-1β [7,8,9], and considering the studies of other research groups [15,18], higher levels of this cytokine are expected in the CRC group. However, different results have reported no difference in IL-1β serum levels between CRC patients and healthy individuals [17]. In an article by Farc et al., a comparison between patients in different disease stages showed significant variation in IL-1β levels; the cytokine concentration decreased between stages I and II, which was followed by an increase in advanced stages (III–IV) [17]. Research by Li et al. showed a significantly higher concentration of IL-1β in the peripheral blood of stage IV patients compared to those in stage II and IIIA, but the data for stage IV patients did not differ markedly from those of the control group and stage I patients [18]. The significantly reduced plasma concentration of IL-1β in our CRC group could be provoked by the general immunological state of the patients. More studies are needed to confirm this hypothesis, but, taking into consideration our immunophenotyping results, it can be concluded that immune response suppression and/or exhaustion are predominant in CRC patients. A factor that contributes to this condition is the chemotherapeutic treatment of the patients.

The populations of NK cells and CD8 T lymphocytes were markedly reduced in the CRC group. This signifies diminished first [20] and second [22] lines of anti-tumor immunity, respectively. In addition, the populations of activated CD25^+^ lymphocytes and CD28^+^ T cells were significantly reduced, suggesting the downregulation of lymphocyte activation [19]. This conclusion is further supported by the detected significantly elevated levels of immune checkpoint molecules CD279, CD274, CD80 and CD86. A tendency for increased CD152 expression on the T cells of CRC patients was also observed. We found a significant correlation between CD8^+^ and CD279^+^ lymphocytes, which suggests cytotoxic lymphocyte exhaustion, as reported previously [22]. Together with the reduced populations of CD8^+^ lymphocytes, these data point to a downregulated anti-tumor immune state of the CRC patients. The roles of CD274 and CD279 interactions have been well defined in CRC [11,29]. Based on our data, the detected elevated populations of CD80^+^ and CD86^+^ lymphocytes in the patients with metastatic disease can also be included in the group of markers for CRC. Elevated CD86 mRNA expression has recently been demonstrated as a potential prognostic biomarker for glioblastoma [30], and significantly enhanced CD80 and CD86 mRNA expression was detected in peripheral blood samples of CRC patients [31]. These data support our findings and stimulate future studies involving early-stage CRC patients to confirm the significance of the suggested panel of biomarkers.

## 5. Conclusions

The expression of CD25, CD28, CD80, CD86, CD274 and CD279 immunoregulatory glycoproteins and the plasma levels of IL-1β, IL-2, IL-15 and TGFβ are altered in stage IV CRC patients receiving chemotherapy. CD8^+^ T lymphocytes and NK cell populations are reduced in CRC patients with metastatic disease compared to healthy individuals. Combined analyses of these molecules could represent a potential panel of biomarkers for colorectal cancer.

## Data Availability

Data are contained within the article or Supplementary Materials.

## Supplementary Materials

The following supporting information can be downloaded at: https://www.mdpi.com/article/10.3390/biom14101314/s1, Table S1: Detailed summary of the data (%) used in the flow cytometry analyses for all participants in the study.

## Abbreviations

CD	cluster of differentiation
CRC	colorectal cancer
CTLA-4	cytotoxic T-lymphocyte-associated protein 4
DPBS	Dulbecco’s phosphate-buffered saline
EDTA	ethylene diamine tetra acetic acid
ELISA	enzyme-linked immunosorbent assay
FACS	fluorescence-activated cell sorting
IFNγ	interferon gamma
IL	interleukin
NK cells	natural killer cells
PD-1	programmed cell death protein 1
TGFβ	transforming growth factor beta

## References

[1] Siegel R.L., Miller K.D., Fuchs H.E., Jemal A. (2022). Cancer statistics, 2022. CA A Cancer J. Clin..

[2] Colorectal Cancer. https://www.who.int/news-room/fact-sheets/detail/colorectal-cancer.

[3] Zhang Y., Wang Y., Zhang B., Li P., Zhao Y. (2023). Methods and biomarkers for early detection, prediction, and diagnosis of colorectal cancer. Biomed. Pharmacother..

[4] Alves Martins B.A., de Bulhoes G.F., Cavalcanti I.N., Martins M.M., de Oliveira P.G., Martins A.M.A. (2019). Biomarkers in Colorectal Cancer: The Role of Translational Proteomics Research. Front. Oncol..

[5] Vacante M., Ciuni R., Basile F., Biondi A. (2020). The Liquid Biopsy in the Management of Colorectal Cancer: An Overview. Biomedicines.

[6] Luo X.J., Zhao Q., Liu J., Zheng J.B., Qiu M.Z., Ju H.Q., Xu R.H. (2021). Novel Genetic and Epigenetic Biomarkers of Prognostic and Predictive Significance in Stage II/III Colorectal Cancer. Mol. Ther. J. Am. Soc. Gene Ther..

[7] Borowczak J., Szczerbowski K., Maniewski M., Kowalewski A., Janiczek-Polewska M., Szylberg A., Marszalek A., Szylberg L. (2022). The Role of Inflammatory Cytokines in the Pathogenesis of Colorectal Carcinoma-Recent Findings and Review. Biomedicines.

[8] Braumüller H., Mauerer B., Andris J., Berlin C., Wieder T., Kesselring R. (2023). The Cytokine Network in Colorectal Cancer: Implications for New Treatment Strategies. Cells.

[9] Maryam S., Krukiewicz K., Haq I.U., Khan A.A., Yahya G., Cavalu S. (2023). Interleukins (Cytokines) as Biomarkers in Colorectal Cancer: Progression, Detection, and Monitoring. J. Clin. Med..

[10] Yamaguchi H., Hsu J.M., Sun L., Wang S.C., Hung M.C. (2024). Advances and prospects of biomarkers for immune checkpoint inhibitors. Cell Rep. Med..

[11] Yang K., Lu R., Mei J., Cao K., Zeng T., Hua Y., Huang X., Li W., Yin Y. (2024). The war between the immune system and the tumor—Using immune biomarkers as tracers. Biomark. Res..

[12] Gunawardene A., Dennett E., Larsen P. (2019). Prognostic value of multiple cytokine analysis in colorectal cancer: A systematic review. J. Gastrointest. Oncol..

[13] Mager L.F., Wasmer M.H., Rau T.T., Krebs P. (2016). Cytokine-Induced Modulation of Colorectal Cancer. Front. Oncol..

[14] Bhat A.A., Nisar S., Singh M., Ashraf B., Masoodi T., Prasad C.P., Sharma A., Maacha S., Karedath T., Hashem S. (2022). Cytokine- and chemokine-induced inflammatory colorectal tumor microenvironment: Emerging avenue for targeted therapy. Cancer Commun..

[15] Czajka-Francuz P., Francuz T., Cison-Jurek S., Czajka A., Fajkis M., Szymczak B., Kozaczka M., Malinowski K.P., Zasada W., Wojnar J. (2020). Serum cytokine profile as a potential prognostic tool in colorectal cancer patients—One center study. Rep. Pract. Oncol. Radiother..

[16] Song X., Traub B., Shi J., Kornmann M. (2021). Possible Roles of Interleukin-4 and -13 and Their Receptors in Gastric and Colon Cancer. Int. J. Mol. Sci..

[17] Farc O., Berindan-Neagoe I., Zaharie F., Budisan L., Zanoaga O., Cristea V. (2022). A role for serum cytokines and cell adhesion molecules in the non-invasive diagnosis of colorectal cancer. Oncol. Lett..

[18] Li W., Chen F., Gao H., Xu Z., Zhou Y., Wang S., Lv Z., Zhang Y., Xu Z., Huo J. (2023). Cytokine concentration in peripheral blood of patients with colorectal cancer. Front. Immunol..

[19] Molon B., Liboni C., Viola A. (2022). CD28 and chemokine receptors: Signalling amplifiers at the immunological synapse. Front. Immunol..

[20] Toffoli E.C., Sheikhi A., Hoppner Y.D., de Kok P., Yazdanpanah-Samani M., Spanholtz J., Verheul H.M.W., van der Vliet H.J., de Gruijl T.D. (2021). Natural Killer Cells and Anti-Cancer Therapies: Reciprocal Effects on Immune Function and Therapeutic Response. Cancers.

[21] Van Den Eeckhout B., Tavernier J., Gerlo S. (2020). Interleukin-1 as Innate Mediator of T Cell Immunity. Front. Immunol..

[22] Zheng Z., Wieder T., Mauerer B., Schafer L., Kesselring R., Braumuller H. (2023). T Cells in Colorectal Cancer: Unravelling the Function of Different T Cell Subsets in the Tumor Microenvironment. Int. J. Mol. Sci..

[23] Long A.G., Lundsmith E.T., Hamilton K.E. (2017). Inflammation and Colorectal Cancer. Curr. Color. Cancer Rep..

[24] Stayoussef M., Weili X., Habel A., Barbirou M., Bedoui S., Attia A., Omrani Y., Zouari K., Maghrebi H., Almawi W.Y. (2024). Altered expression of cytokines, chemokines, growth factors, and soluble receptors in patients with colorectal cancer, and correlation with treatment outcome. Cancer Immunol. Immunother..

[25] Urbiola-Salvador V., Jablonska A., Miroszewska D., Huang Q., Duzowska K., Drezek-Chyla K., Zdrenka M., Srutek E., Szylberg L., Jankowski M. (2023). Plasma protein changes reflect colorectal cancer development and associated inflammation. Front. Oncol..

[26] Berghella A.M., Contasta I., Pellegrini P., Del Beato T., Adorno D. (2002). Peripheral blood immunological parameters for use as markers of pre-invasive to invasive colorectal cancer. Cancer Biother. Radiopharm..

[27] Wang H.P., Wang Y.Y., Pan J., Cen R., Cai Y.K. (2014). Evaluation of specific fecal protein biochips for the diagnosis of colorectal cancer. World J. Gastroenterol..

[28] Abbas A.K. (2020). The Surprising Story of IL-2: From Experimental Models to Clinical Application. Am. J. Pathol..

[29] Chen X., Chen L.J., Peng X.F., Deng L., Wang Y., Li J.J., Guo D.L., Niu X.H. (2024). Anti-PD-1/PD-L1 therapy for colorectal cancer: Clinical implications and future considerations. Transl. Oncol..

[30] Ahmed M.H., Hernandez-Verdin I., Bielle F., Verreault M., Lerond J., Alentorn A., Sanson M., Idbaih A. (2023). Expression and Prognostic Value of CD80 and CD86 in the Tumor Microenvironment of Newly Diagnosed Glioblastoma. Can. J. Neurol. Sci..

[31] Ismael A., Jabbar S. (2024). Expressions of CD80 and CD86 in Cancer Patients and Its Prognostic Significance. J. Pioneer. Med. Sci..

