# Supplementary material for: Some Glycoproteins Expressed on the Surface of Immune Cells and Cytokine Plasma Levels Can Be Used as Potential Biomarkers in Patients with Colorectal Cancer
Source: Biomolecules. 2024 Oct 16;14(10):1314. doi: 10.3390/biom14101314 (PMC11505977; doi:10.3390/biom14101314)
Supplement: Supplementary file 1 [file biomolecules-14-01314-s001.zip › biomolecules-3211464-supplementary.pdf]

Table S1. Detailed summary of the data (%) used in the flow cytometry analyses for all participants in the study.

Samples	T cells (CD3+)	CD28	CD4	CD8	CD25	CD152	CD279	B cells (CD20)	CD80	CD86	CD273	CD274	NK (CD56+/CD16+)	NK (CD56+/CD16-)
**Control**	45,100	70,50	13,60	7,87	0,37	0,13	0,028	5,68	6,57	0,000	5,26	0,260	9,42	18,10
**Control**	43,500	95,80	12,00	3,99	0,45	1,76	0,026	0,74	1,84	0,014	0,71	0,041	10,60	10,60
**Control**	36,400	85,10	29,00	9,18	0,24	0,65	0,023	1,53	1,85	0,008	1,49	0,150	2,46	4,52
**Control**	53,400	83,90	24,60	8,30	0,46	0,85	0,028	0,72	0,96	0,016	0,67	0,083	1,85	4,02
**Control**	23,900	85,80	37,30	15,00	3,13	0,62	0,007	1,29	1,75	0,017	1,29	0,100	16,10	26,10
**Control**	40,600	85,40	63,90	21,10	22,80	14,70	0,010	0,49	1,32	0,004	0,57	0,210	16,90	25,60
**Control**	32,700	88,90	26,90	12,70	0,76	0,52	0,007	2,17	3,32	0,002	1,96	0,120	9,29	44,90
**Control**	36,200	91,10	51,40	20,10	2,48	0,77	0,037	3,26	5,31	0,016	3,24	0,260	12,90	40,00
**Control**	5,600	85,60	60,90	22,10	2,65	1,20	0,000	1,10	1,15	0,001	1,06	0,075	0,57	2,37
**Control**	3,330	88,50	42,20	29,40	1,15	2,44	0,070	2,09	1,95	0,006	2,06	0,100	8,23	22,00
**Control**	1,960	91,50	13,80	5,22	0,25	1,79	0,060	4,21	5,67	0,000	4,14	0,340	7,43	21,60
**Control**	8,830	91,20	7,63	4,03	0,10	1,21	0,016	2,86	4,48	0,005	2,55	0,110	12,40	25,10
**Control**	24,000	86,20	10,40	5,75	0,85	0,69	0,007	7,24	3,57	0,010	6,86	0,230	7,47	42,70
**Control**	35,600	86,70	3,92	2,03	0,14	9,73	0,051	2,03	10,70	0,000	1,95	0,480	1,20	3,63
**Control**	38,500	93,70	3,24	1,61	0,12	0,63	0,011	6,14	3,94	0,019	4,39	0,280	4,16	18,10
**Control**	9,000	98,00	15,00	4,40	3,06	0,41	0,014	1,79	1,75	0,025	1,76	0,310	8,69	31,70
**Control**	52,500	93,00	5,55	2,21	2,03	0,62	0,004	8,63	10,90	0,002	8,02	0,370	11,90	50,30
**Control**	64,400	88,50	35,30	11,60	0,55	0,45	0,025	10,20	6,04	0,007	9,96	0,830	9,83	33,50
**Control**	47,600	81,20	12,60	9,32	0,98	1,39	0,022	6,69	9,02	0,010	6,39	0,730	0,91	3,59
**Control**	39,900	78,50	10,40	8,41	0,58	3,25	0,036	12,70	8,41	0,002	12,10	1,790	5,40	18,40
**Control**	60,900	93,70	22,60	6,76	0,42	0,88	0,007	13,30	7,93	0,004	12,40	0,550	12,90	27,10
**CRC**	7,670	76,60	8,47	4,09	0,22	0,15	2,610	0,57	0,28	0,200	0,34	0,340	1,07	6,53
**CRC**	15,300	77,70	14,50	5,67	0,30	0,32	1,620	1,42	0,87	0,120	1,30	0,560	2,13	11,80
**CRC**	64,000	62,00	60,60	25,80	0,75	0,06	5,280	6,38	19,10	0,005	6,80	0,650	4,88	48,70
**CRC**	31,500	85,10	52,10	33,00	0,20	0,22	1,850	8,25	10,40	0,005	8,53	0,290	1,40	35,10
**CRC**	42,200	96,60	33,40	4,95	0,71	0,12	0,660	1,27	12,90	0,160	1,87	1,470	2,02	40,80
**CRC**	4,970	41,20	59,60	8,47	0,78	0,15	12,600	5,88	18,00	0,016	6,43	0,640	3,20	33,70
**CRC**	12,700	86,10	4,37	2,36	0,27	0,00	4,340	1,63	3,40	0,046	1,82	0,700	1,35	20,60
**CRC**	34,300	57,50	6,22	2,51	0,56	0,09	0,500	1,44	1,23	0,043	1,66	0,780	2,27	21,10
**CRC**	43,800	84,90	28,20	14,70	1,23	0,10	1,380	5,11	6,93	0,041	5,84	1,170	7,06	30,20
**CRC**	8,510	50,00	54,30	0,03	0,17	50,00	0,000	2,97	17,20	0,000	3,58	3,580	0,79	25,60
**CRC**	21,500	82,40	67,40	0,00	0,07	29,40	0,000	3,42	15,40	0,010	4,74	2,380	0,92	17,60
**CRC**	45,200	72,90	49,20	0,01	0,26	12,90	0,000	4,15	11,20	0,008	6,30	1,370	0,80	22,70
**CRC**	66,800	81,90	60,70	0,04	0,55	1,67	0,920	5,16	14,90	0,000	6,18	1,680	3,27	44,00
**CRC**	26,600	61,30	22,60	0,00	0,29	2,17	0,460	5,01	13,90	0,068	6,48	2,270	4,68	21,80
**CRC**	15,300	70,50	23,90	0,00	0,18	3,29	0,650	2,21	13,40	0,059	3,01	1,300	9,44	23,30
**CRC**	18,300	74,50	13,60	7,87	0,37	0,14	16,300	1,17	11,80	0,007	1,56	0,610	1,99	28,40
**CRC**	35,900	76,40	29,70	0,00	0,32	0,22	15,200	2,97	24,70	0,012	3,40	0,960	4,94	59,50
**CRC**	4,800	80,30	10,60	3,36	0,14	2,34	2,300	5,68	6,57	0,000	5,26	0,260	0,42	3,97
**CRC**	24,200	86,60	25,10	7,72	0,16	0,21	5,990	1,19	3,58	0,015	1,43	0,480	0,10	4,42
**CRC**	15,000	31,20	5,56	5,92	0,08	0,74	7,300	0,98	3,12	0,019	1,22	0,600	0,10	8,65
